# Focal adhesion kinase: from biological functions to therapeutic strategies

**DOI:** 10.1186/s40164-023-00446-7

**Published:** 2023-09-25

**Authors:** Ximin Tan, Yuheng Yan, Bin Song, Shuangli Zhu, Qi Mei, Kongming Wu

**Affiliations:** 1 Department of Oncology, Tongji Hospital of Tongji Medical College, Huazhong University of Science and Technology, Wuhan, 430030 China; 2 Cancer Center, Shanxi Bethune Hospital, Shanxi Academy of Medical Science, Tongji Shanxi Hospital, Third Hospital of Shanxi Medical University, Taiyuan, 030032 China; 3 Cancer Center, Tongji Hospital of Tongji Medical College, Huazhong University of Science and Technology, Wuhan, 430030 China

**Keywords:** Focal adhesion kinase, Immunotherapy, Combination therapy, Tumor microenvironment

## Abstract

Focal adhesion kinase (FAK), a nonreceptor cytoplasmic tyrosine kinase, is a vital participant in primary cellular functions, such as proliferation, survival, migration, and invasion. In addition, FAK regulates cancer stem cell activities and contributes to the formation of the tumor microenvironment (TME). Importantly, increased FAK expression and activity are strongly associated with unfavorable clinical outcomes and metastatic characteristics in numerous tumors. In vitro and in vivo studies have demonstrated that modulating FAK activity by application of FAK inhibitors alone or in combination treatment regimens could be effective for cancer therapy. Based on these findings, several agents targeting FAK have been exploited in diverse preclinical tumor models. This article briefly describes the structure and function of FAK, as well as research progress on FAK inhibitors in combination therapies. We also discuss the challenges and future directions regarding anti-FAK combination therapies.

## Background

Focal adhesion kinase is a tyrosine kinase composed of 1052 amino acids with a molecular weight of 125kD [1]. FAK is a crucial regulator of vital cellular processes, including cell adhesion [2], migration [3], proliferation [4], and survival [5]. Such processes have significant implications for the development and progression of cancer. Moreover, multiple studies have confirmed FAK upregulation in a diverse range of human malignancies, including colorectal, lung, ovarian, neck, bladder, breast, and esophageal cancers [6]. In addition, by promoting tumor angiogenesis, epithelial-mesenchymal transformation, cancer stemness, and immunomodulatory capacity [7–9], FAK significantly contributes to malignant progression. The available evidence suggests that FAK represents a promising target for cancer therapy. This article aims to provide an overview of the significant impact of FAK on both cancer cells and cancer-associated cells within tumors while also offering a comprehensive discussion of the recent advances made in developing therapeutic agents that target FAK and the combination of these agents with other approaches.

## FAK structure and activation

### Molecular structure of FAK

**Fig. 1:**
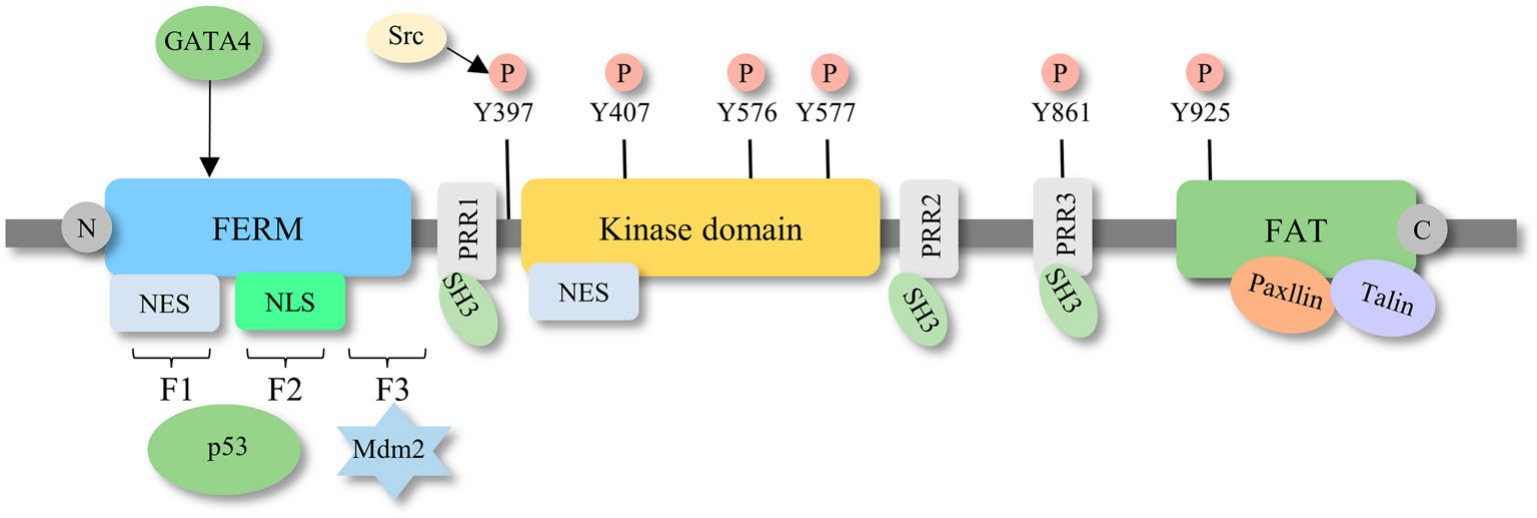
Schematic diagram of the structural domain of FAK. FAK comprises three primary components, including a central kinase domain, a FERM domain on the N-terminal side, and a FAT domain on the C-terminal side. The kinase domain, which is crucial for catalytic activity, is flanked by three proline-rich regions that are responsible for protein‒protein interactions. The FAK phosphorylation sites and important binding proteins that regulate FAK activity and downstream signaling are highlighted in the diagram

FAK is composed of three distinct domains: a four-point-one-ezrin-radixin-moesin (FERM) domain, a kinase domain, and a C-terminal focal adhesion-targeting (FAT) domain [10]. To target different sites on FAK, inhibitors specific to each domain have been developed (Fig. 1). The FERM domain is further divided into F1, F2, and F3 subdomains. F1 contains a nuclear export sequence, and F2 contains a nuclear localization sequence [11]. As the names indicate, these sequences are important for the nuclear transport of FAK. Over the past few years, research has emphasized the significance of nuclear FAK in the regulation of gene expression; it interacts with distinct E3 ligases to induce the degradation of transcription factors [12]. For example, the F1 and F2 lobes interact with p53, followed by the combination of the F3 lobe with murine double minute2 (Mdm2) and subsequent p53 ubiquitination and degradation, thus facilitating cancer cell proliferation and inhibiting apoptosis [13]. Nuclear FAK also alters expression of GATA4 and IL-33, suppressing the inflammatory response and inducing immune escape [14–17]. Aside from controlling FAK transportation from the cytoplasm to the nucleus, the FERM domain also has a crucial function in triggering the activation of FAK. For example, the link between the FERM domain and the kinase domain prevents FAK activation by blocking the autophosphorylation of Y397, which is the sole autophosphorylation site of FAK [18]. The FERM domain contains binding sites for numerous proteins, such as integrin [19], growth factor receptors [20, 21], and G-protein coupled estrogen receptors [22], and such interactions trigger downstream signaling cascades and induces FAK activation. The KAKTLRK sequence, which is in the F2 lobe, also participates in FAK activation [23].

The bilobal central kinase domain, which contains catalytic sites and ATP-binding sites, is highly homological with other tyrosine kinases, particularly proline-rich tyrosine kinase 2 (Pyk2), and serves as the major component for FAK enzymatic activity [24]. Nuclear export signal 2 (NES2), which is regarded as the only biologically active nuclear export signal (NES) exerting nuclear export activities, is located in the central kinase domain [25].

The C-terminal region, which resembles a bundle composed of four helices, is primarily comprised of the FAT domain and has a significant role in controlling the activation of FAK. Through its interaction with proteins associated with focal adhesions such as paxillin and talin, the FAT domain prompts the recruitment and activation of FAK at the site of focal adhesions [26, 27]. This interaction is pivotal for FA assembly and turnover, which influences cell motility. In contrast, other studies have indicated that the FAT domain functions as an inhibitor of FAK activation by competing with the FERM domain for the binding of specific intracellular receptors and inducing dephosphorylation [28].

In addition to the three major domains, there are some special residues regulating FAK activation and function. FAK contains three proline-rich regions (PRRs), PRR1 localizes in the N-terminal domain, and PRR2/3 localizes in the C-terminal domain next to the FAT region. PRRs attach to proteins that contain Src homology 3 (SH3) domains, including small GTPases and p130Cas, to regulate kinase activity and support the cytoskeleton [29]. Additionally, there are at minimum six tyrosine residues that undergo phosphorylation throughout the entire region: Y397, Y407, Y576, Y577, Y861, and Y925. The autophosphorylation of Y397, which is situated at the N-terminus of the FERM structural domain, is vital for the activation of FAK [30]. Phosphorylated Y397 provides a binding site for Src-family kinases and other proteins containing the SH2 domain [31], which also interact with other tyrosine residues and are pivotal for FAK catalytic activity. Located in the activation loop of the kinase domain [32, 33], Y576 and Y577 positively regulate FAK kinase function, while another kinase domain-located tyrosine phosphorylation site, Y407, inhibits FAK activity. Additionally, Y861 and Y925 are in the C-terminal domains. Phosphorylated Y925 interacts with the SH2 domain of the adaptor protein GRB2 to trigger downstream Ras/MAPK signaling and induce integrin internalization and focal adhesion disassembly [34]. This FAK-GRB2-MAPK linkage was also demonstrated to be essential for tumor angiogenesis [35].

### The mechanism and regulation of FAK activation

**Fig. 2:**
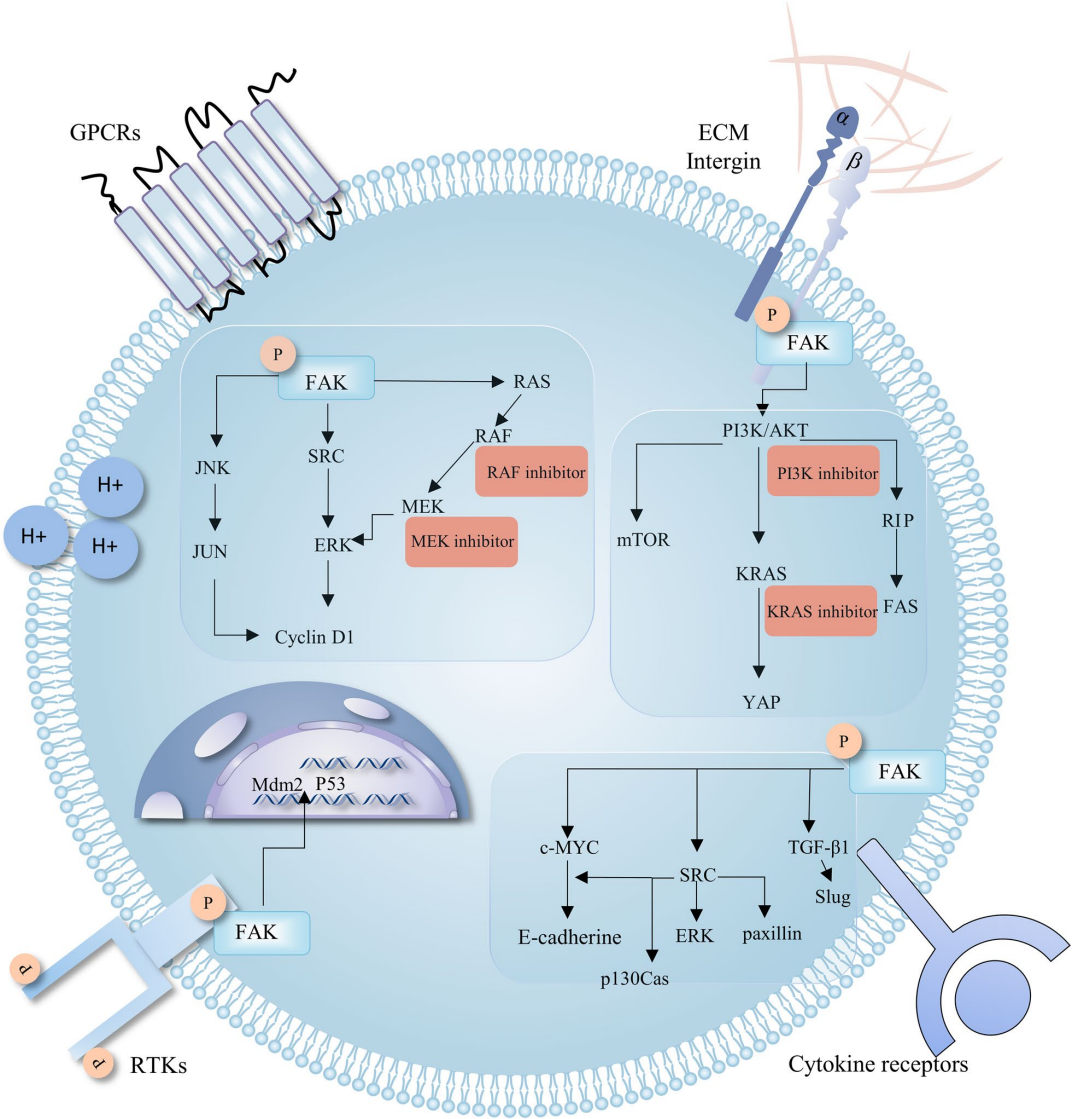
FAK-mediated signaling cascades involved in tumor progression. FAK activation is multifaceted and can be mediated by various factors, such as integrins, receptor tyrosine kinases (RTKs), mechanical stimuli, cytokines, G-protein-coupled receptors (GPCRs), and a change in intracellular pH (H+). Upon phosphorylation, FAK may induce the activation of different transduction pathways, including RAS/RAF/ERK, JNK, YAP, and PI3K/AKT/mTOR signaling. This process can lead to the regulation of relevant oncogenes, which in turn supports cancer cell survival. FAK also exerts nuclear functions, acting as a scaffold for p53 and Mdm2 while also promoting the polyubiquitination and degradation of p53. In doing so, FAK again promotes resistance to apoptosis. As shown in the diagram, the highlighted red boxes indicate targets of interest for the development of combination therapy using FAK inhibitors

FAK is a vital mediator in the transmission of signals from the extracellular matrix (ECM) to the cell cytoplasm. It regulates pivotal cellular functions, including cell survival, proliferation, migration, and invasion (Fig. 2). In response to upstream stimuli, FAK initiates downstream signaling cascades, thereby prompting a series of events. Additionally, FAK is versatile as a signaling molecule because it can demonstrate kinase-dependent or kinase-independent activity [36]. Stimuli, integrin signaling activation, in particular, disrupts the inhibitory interaction between FERM and kinase domains, which leads to FAK dimerization and causes subsequent Y397 autophosphorylation [28]. The extensively phosphorylated Y397 has high affinity for the SH2 domain of kinases belonging to the Src family. When this interaction occurs, these kinases bind to and phosphorylate Y576 and Y577, which are positioned in the activation loop, to achieve complete activation of FAK and endow it with its enzymatic function [24].

The activation of FAK is regulated by various internal and external factors, which mainly target the FERM domain to induce conformational changes, thus relieving the autoinhibitory structure between the FERM and the kinase domain and stimulating FAK activation. In addition to integrin, the uncovered binding partners of the FERM domain include extracellular matrix, phosphoinositide lipids, diacylglycerol kinase α, serine/threonine kinase PKCθ, and membrane-associated proteins such as tetraspanin transmembrane 4 L6 family member 5 (TM4LFM5) and EMP2 [24, 28, 37–39]. Intriguingly, glutathione peroxidase-1 can bind to FAK and prevent H_2_O_2_-induced oxidative inactivation of FAK [40]. Furthermore, growth factor receptors, including the Met receptor for hepatocyte growth factor, epidermal growth factor receptor, and platelet-derived growth factor receptor, enable conformational changes in the FERM domain by phosphorylating either the Y397 or Y197 sites [41, 42]. Other stimuli, such as elevated intracellular pH and increased matrix stiffness or forces, which occur during cancer progression, also trigger Y397 phosphorylation and FAK activation [43, 44]. Recently, it was revealed that FAK expression and activation are epigenetically regulated [45]. For example, microRNA miR-15b-5p inhibits FAK expression by binding to the 3′UTR of FAK mRNA. Moreover, intercellular adhesion molecule-1 suppresses miR-15b-5p activity and stimulates endothelial cell proliferation and migration [46]. The interaction between long noncoding RNA (lncRNA) MIR4435‑2HG and ganglioside synthesis enzyme ST8SIA1 induces the activation of FAK and downstream AKT/β‑catenin signaling, thus promoting prostate cancer cell viability [45]. Epidermal growth factor and IL-6, which are highly activated in glioblastoma, also initiate FAK activation [47].

## The functions of FAK on tumor cells

### Tumor cell proliferation, apoptosis and survival

The involvement of FAK in tumor growth has been extensively investigated in human breast cancer. The PI3K/AKT/mTOR signaling pathway has been widely recognized as one of the most commonly disrupted pathways in cancer [48, 49], and is correlated with FAK-mediated tumor cell growth. The ablation of FAK reduced Wnt1-driven basal-like breast cancer growth and promotes apoptosis by downregulating AKT-mTOR signaling [50]. In addition, activation of FAK by insulin-like growth factor-1 (IGF-1) and its receptor system (IGF-1R) initiates the PI3K-AKT-YAP (yes-associated protein/yes-related protein) signaling cascade, which modulates the expression of genes targeted by YAP. This mechanism is implicated in the expansion of aggressive triple-negative breast cancer cells [51]. A similar result was observed in intrahepatic cholangiocarcinoma (iCCA) [52]. FAK activation, which is required for Y357 phosphorylation of YAP, strongly promotes AKT/YAP-driven mouse iCCA initiation [52].

FAK was also confirmed to stimulate cell cycle progression and promote cancer proliferation by targeting various cyclins and cyclin-dependent kinase (CDK) inhibitors [53]. Among them, cyclin D1, together with key CDK inhibitors p21 and p27, are the most extensively studied downstream targets of FAK, regulating the cell cycle transition from G1 to S phase. It was reported that knockdown of Mucin-like 1 (MUCL1) in HER2-amplified breast cancer resulted in FAK/Jun NH2-terminal kinase (JNK) signaling blockade and subsequent G1/S phase arrest, which was mediated by decreased cyclin D levels as well as increased p21 and p27 levels [54]. A mechanistic study revealed that integrin signaling through FAK activated the ERK pathway, which stimulated the transcriptional activation of cyclin D [55]. In addition to p21 and p27, it was also demonstrated that FAK ablation in glioblastoma repressed the expression of the autophagy cargo receptor p62/SQSTM-1, the inhibition of which post transcriptionally upregulated p27 expression to mediate G1 phase arrest and induced a cell senescence-like state [56]. Moreover, p62 synergized with its downstream target SKP2 to inhibit p21 and p27 activity. Nevertheless, contradictory results were observed in vascular smooth muscle cells: SKP2 was degraded by nuclear FAK to inhibit cell proliferation by promoting p21 and p27 expression [57]. It has been acknowledged that nuclear FAK acts as a scaffold for protein interactions and regulates specific gene transcription. In skin squamous cell carcinoma, nuclear FAK was reported to interact with runt-related transcription factor 1 (Runx1) and recruit Runx1 regulatory proteins such as sin3a to inhibit the transcription of insulin-like growth factor binding protein 3, which induces cell cycle arrest at the G1 phase by suppressing the expression of cyclins and CDKs and increasing p21 expression [58]. Likewise, the nuclear activation of FAK in colon cancer cells by fibrin resulted in a decline in p53, along with its subsequent targets, such as 14-3-3σ and p21, ultimately stimulating cell proliferation while repressing senescence [59].

FAK plays a vital role in sustaining cancer cell survival and regulating cell apoptosis [60–63], anoikis [64–68], autophagy [68], and senescence [69]. The disruption of FAK-mediated signaling in breast cancer cells stimulated the Fas-associated death domain (FADD) and caspase-8 apoptotic pathways, which induced cell apoptosis and inhibited anchorage-independent survival [70]. Additionally, components of death receptor pathways, including the FAK-binding partner death domain kinase receptor-interacting protein (RIP), are involved in this process [61]. The antiapoptotic effect of FAK was further confirmed to be associated with FAK-dependent activation of the phosphatidylinositide 3′-OH-kinase-AKT survival pathway, concomitant with the subsequent stimulation of NF-kB and inhibitor-of-apoptosis proteins [71]. Moreover, nuclear FAK also plays an important role in regulating cancer cell apoptosis. Nuclear FAK interacts with the N-terminal transactivation domain of p53 through its N-terminal fragment; this attenuates p53 transcriptional activity and inhibits p53-mediated apoptosis to promote cell survival [72]. Anoikis, a distinct type of apoptosis that occurs in normal epithelial and endothelial cells, is also negatively regulated by FAK signaling In human breast cancer, the mechanism of FAK-induced cell resistance to anoikis was reported to be correlated with the increased activity of NF-kB, which is induced by the functional interaction between the N-terminal domain of FAK and TRAF2, a RING finger adaptor protein [65]. Notably, integrin endocytosis has gradually been uncovered to be critical for FAK activation depending on endosome antigen-1 and small GTPase Rab21, and FAK activation ultimately promotes anoikis resistance and anchorage-independent cell growth [66, 67].

### Cell migration and invasion

Integrin aggregation in the ECM plays a crucial role in inducing FAK signaling, which is fundamental for cell motility and cytoskeletal reorganization. Chemotactic signals activate integrins, leading to FAK activation and the formation of focal adhesion complexes, which subsequently trigger the polymerization of actin filaments toward the cytoplasmic membrane [73, 74]. The FAK/Src complex and kinase activity lead to p130Cas phosphorylation, promoting the formation of Cas/Crk complexes, which significantly influence cell migration [75]. Additionally, MLCK-mediated focal adhesion disassembly and JNK-mediated paxillin phosphorylation promote cytoskeleton reorganization [76, 77]. FAK’s associations with PI3-kinase and/or Grb7 govern intracellular signaling pathways correlated with cellular mobility [78, 79]. Furthermore, integrin-mediated FAK signaling critically controls adhesion dynamics during cell migration. The formation of FAK/Src complexes at focal adhesion sites enhances ERK2 activity, resulting in the activation of Calpain 2 [80–82]. FAK’s effects on small GTPases impact cytoskeletal reorganization and adhesion stabilization [83]. RhoA, Rac1, and Cdc42, among small GTPases, play a crucial role in cytoskeletal reorganization and tumorigenesis [84, 85]. In this regard, RhoA influences cell‒cell or cell–ECM associations by inducing shifts within the cytoskeleton, while Rac1 initiates actin polymerization, enabling membrane folding, whereas Cdc42 initiates actin filament production in the generation of filopodia [86–88]. These investigations present compelling proof that the expression of FAK and the activation of FAK signaling pathways are mainly mediated by Rho GTPases, indicating the indispensable role of FAK in cytoskeletal reorganization.

### Epithelial–mesenchymal transformation

Epithelial–mesenchymal transition (EMT) is a physiological process in normal embryonic development and tissue regeneration. However, aberrant reactivation of EMT is associated with malignant properties of tumor cells, including migration, invasiveness, increased tumor stemness, and resistance to chemotherapy and immunotherapy [88, 89]. Multiple studies have established the role of FAK in promoting EMT and increasing cell invasion and metastasis [90, 91]. EMT is regulated by FAK-mediated alterations in E-cadherin expression, a key molecule in the process [92–94]. Evidence provided by Gayrard et al. illustrates that SRC-FAK-mediated reconstruction of actomyosin results in the loosening of E-cadherin junctions without disrupting those involving β-associated proteins [94]. Avizienyte’s team confirmed the importance of FAK phosphorylation in the decrease in E-cadherin induced by Src in colon cancer cells [91]. The investigation carried out by Hauck’s team suggests that restraining FAK function restricts cell invasion stimulated by Src and obstructs the metastasis and invasion aimed by FAK-targeted drugs [95]. The downregulation of KIF26A clearly enhances EMT and decreases E-cadherin expression by augmenting the binding of c-MYC to the promoter section of FAK [96]. Slug expression, which balances EMT and cellular migration, is triggered by TGF-β1 in squamous cell carcinoma cells. However, when FAK inhibitors are administered, such an effect is alleviated [97]. These findings underline the crucial role of FAK in EMT, invasion, and metastasis. Nonetheless, additional research is required to clarify the downstream molecular mechanisms by which FAK regulates EMT. These mechanisms include E-cadherin-mediated cell‒cell adhesion, integrin-ECM-based adhesion, and the collaboration of both these mechanisms.

### FAK in cancer stem cells

In various tumor types, FAK has been found to contribute to the activities of cancer stem cells (CSCs), particularly in breast cancer [98, 99]. Loss of FAK leads to a decrease in mammary cancer stem cells (MaCSCs) and suppresses their protumorigenic functions [100]. FAK facilitates the formation of a ternary complex with connexin and NANOG, which sustains CSC self-renewal and maintenance in triple-negative breast cancer [101]. By interrupting the interaction between FAK and endophilin A2, the stem-like population, gene signature, self-renewal, and tumorigenicity of mammary CSCs can be suppressed [102]. In triple-negative breast cancer (TNBC), inhibiting FAK genetically or with drugs decreases anchorage-independent spheroid cell growth, reduces chemotherapy-dependent CSC enrichment, and delays metastatic outgrowth [99, 103]. A recent study reported that inhibiting FAK, a protein found in head and neck squamous cell carcinoma (HNSCC), could significantly reduce the expression of stem cell markers, including Oct4, Sox2, and Nanog, leading to a decrease in cell self-renewal [104]. Additionally, FAK-mediated signaling pathways have been discovered to play a crucial role in regulating CSC properties in esophageal squamous cell carcinoma [105].

## The effects of FAK on tumor-associated cells

### Immune cells

**Fig. 3:**
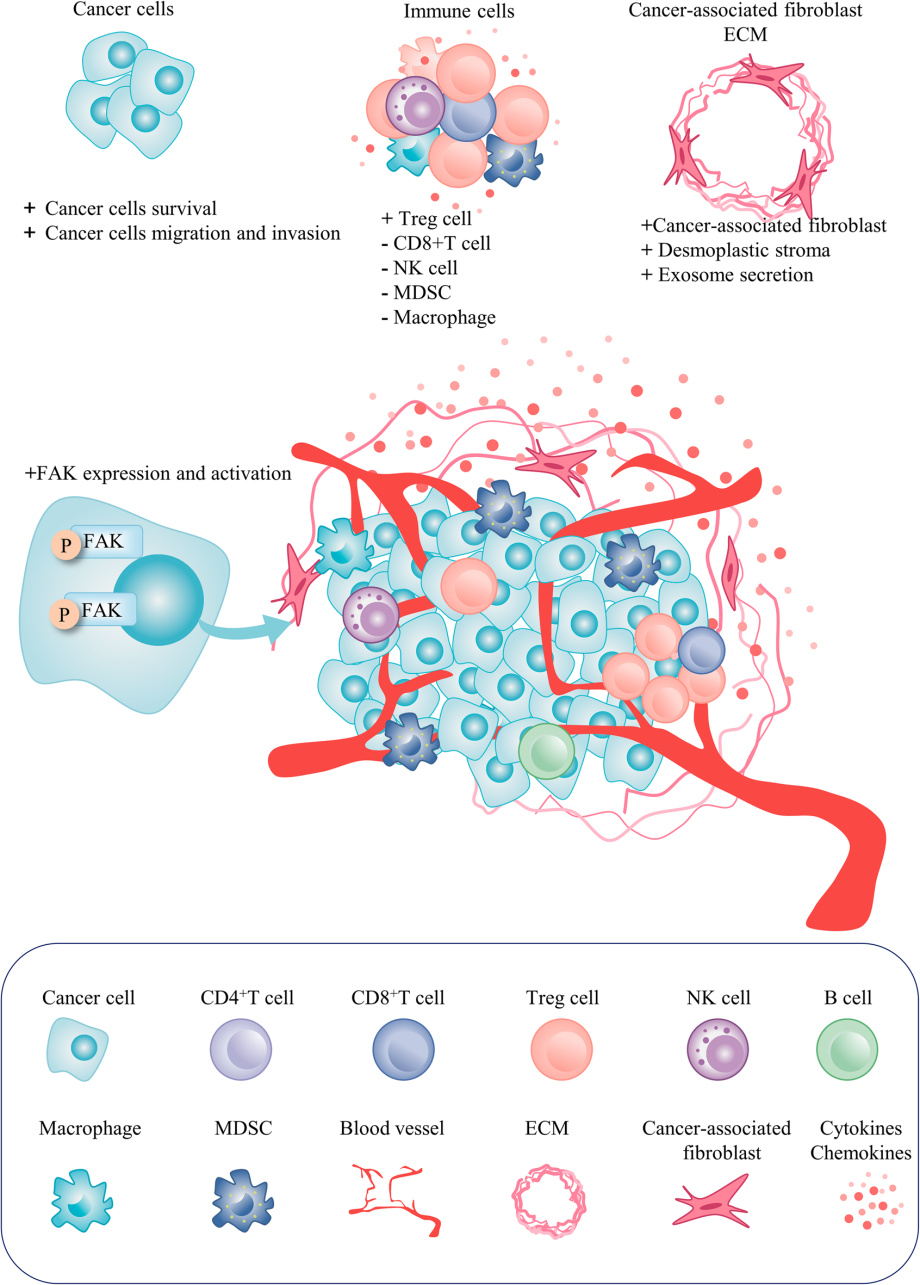
The role of FAK in the tumor microenvironment. FAK not only maintains cancer malignancy but also influences remodeling of the immune microenvironment. For example, FAK can recruit immune cells, cancer-associated fibroblasts, and endothelial cells and even remodel the extracellular matrix. Preclinical studies support the importance of FAK inhibitors in combination with other immunotherapies, and relevant clinical trials are in progress, demonstrating the importance of FAK in oncology treatment

Tumor-associated macrophage and regulatory T cells (Treg) are major inhibitory cells for anti-cancer immune response [106–108]. The importance of FAK expression in the regulation of the tumor environment has been emphasized in current research (Fig. 3). Enhancement of the expression of several chemokines occurs due to the elevation of FAK levels in tumors, which promotes TME remodeling by recruiting immunosuppressive cells and secreting cytokines [109]. FAK inhibitors have been shown to suppress leucocyte and macrophage infiltration and the growth of breast cancer [110, 111] and pancreatic ductal adenocarcinoma (PDAC) tumors in mouse models [112]. FAK signaling enhances the expression of homing signals such as CCL5, CCL7, CXCL10, and TGFβ2 [113], which play a crucial role in recruiting Tregs [114]. FAK contributes to the increased expression of IL-33 [115], an alarmin cytokine, produced by stromal and epithelial cells, which binds to ST2L on immune cells and enhances the transcription of chemokine genes such as CCL5 [116]. The increased production of CCL5 leads to the recruitment of Tregs and other immune cells, promoting immunosuppression. However, recruited Tregs promote tumor survival by depleting CD8+ cytotoxic cells [117]. CD28 is a costimulatory molecule on T cells that enhances T-cell activation and proliferation. Amplification of CD28+ T cells within the TME can enhance their antitumor effects and facilitate tumor cell elimination. FAK depletion can lead to tumor regression by increasing the number of CD28+ T cells in the TME [118]. FAK overexpression slows tumor growth and promotes natural killer cell infiltrations while FAK knockdown promotes tumor growth and suppresses natural killer cell infiltrations [119].

### Cancer-associated fibroblasts (CAFs)

In the TME, CAFs are key stromal cells that play a critical role in tumor cell initiation, survival, proliferation, and metastasis through the secretion of various cytokines, growth factors, hormones, and ECM proteins [120]. For example, increased lumican expression in gastric CAFs promotes FAK activation via β1 integrin, promoting the invasion of gastric cancer cells [121]. PDAC cells activate CAFs and promote cancer stemness through increased expression of type I collagen via β1 integrin-FAK signaling [122]. Inhibition of FAK reduces CAF recruitment and TME fibrosis [123]. This reduces the stemness of PDAC cells [124] and suppresses breast cancer metastasis while increasing the levels of tumor suppressor microRNAs in exosomes [125]. Emerging evidence highlights the pivotal role of CAFs in governing tumor metabolic processes via FAK-regulated pathways. Notably, breast and pancreatic cancer patients exhibiting diminished FAK expression experience a significant decline in overall survival. Furthermore, experimental studies using mouse models have demonstrated that the depletion of FAK in CAFs actively promotes tumor growth. Mechanistically, this phenomenon can be attributed to the activation of protein kinase A within CAFs, resulting from the deficiency of FAK. Consequently, this activation leads to a pronounced enhancement of glycolysis in tumor cells [126]. The remodeling of the TME is facilitated by FAK, which functions as a crucial regulator in the TME. Therefore, a comprehensive understanding of the impact of FAK on tumor progression and TME remodeling could reveal new opportunities for cancer therapy.

### Endothelial cells (ECs)

Integrins and growth factor receptors mediate the signals involved in angiogenesis. FAK is activated by integrin-mediated cell adhesion and associates with several proteins that contain the SH2 structural domain, including Src, Grb7, the p85 subunit of PI3K, and phospholipase C-g [127]. FAK interacts with epidermal growth factor receptor (EGFR), vascular endothelial growth factor receptor (VEGFR), and platelet-derived growth factor receptor (PDGFR) via its N-terminus, and VEGF induces FAK phosphorylation through VEGFR activation, promoting angiogenesis [128]. When platelet-derived growth factor (PDGF) binds to PDGFR, it phosphorylates FAK, which activates endothelial cells, stromal cells, and CEPs, leading to matrix metalloproteinase-mediated breakdown of the ECM and angiogenesis [129]. FAK inhibition is a promising anticancer treatment strategy to hinder cell migration, invasion, proliferation, and angiogenesis. Inhibition of the phosphorylation of vascular endothelial growth factor receptor 2 (VEGFR2), Src, and FAK through Sema3A led to substantial reductions in tumor growth and angiogenesis in tongue SSC-9 cells, illustrating the potential of this approach for cancer therapy [63]. TAE226, a potent FAK inhibitor, effectively suppressed the growth of OSCC xenografts and angiogenesis in mice [130]. Moreover, FRNK, a negative inhibitor of FAK, was revealed to impede FAK phosphorylation, thereby reducing EGF-induced MMP-9 expression and ultimately hindering the invasion of follicular thyroid cancer cells [131]. These collective findings affirm the pivotal role of FAK in inducing cell invasion and angiogenesis and its potential as an attractive target for antiangiogenic therapy in cancer treatment.

## Targeting FAK in combination therapies

FAK is considered a potential target for effective cancer therapy, and its key role in various types of cancer has been well established. Recently, FAK inhibitors have gained attention as novel and promising combination therapy partners (Fig. 3). Presented here is a summary of preclinical (Table 1) and clinical trials (Table 2) concerning FAK inhibitors. The primary focus is on combination trials incorporating FAK as a therapeutic target (Table 3) to assess its efficacy and its contribution to combination therapy.

**Table 1:** Summary of FAK inhibitors in preclinical trials

Name	Alternative names	Target(s)	Cancer types	In vitro	In vivo	References
VS-6062	PF-562, 271	FAK, Pyk2	Glioma	+	+	[175]
			Colon cancer	+	+	[175]
			Breast cancer	+	+	[175]
			Pancreatic cancer	+	+	[112, 175]
			Prostate cancer	+	+	[175–177]
			Lung cancer	+	+	[175, 178]
			Hepatocellular cancer	+	+	[179]
			Thyroid tumor	+	+	[180]
			B16	−	+	[181]
			Ovarian cancer	+	+	[182]
			Ewing sarcoma	+	+	[183]
VS-6063	Defactinib, PF-04554878	FAK, Pyk2	Pancreatic cancer	+	+	[156, 184]
			Lung cancer	+	+	[5, 164, 165]
			Prostate cancer	+	+	[188]
			Ovarian cancer	+	+	[189, 190]
			Esophageal cancer	+	+	[191]
			Endometrial cancer	+	+	[192]
BI-853520	IN10018, ifebemtinib	FAK	Breast cancer	+	+	[193]
			KRAS G12C mutant cancer	+	+	[145]
			Pancreatic cancer	+	+	[162]
			Ovarian cancer	+	+	[145, 194]
			Prostate cancer	+	+	[195]
PF-573228	PF-228	FAK	Pancreatic cancer	+	+	[196]
			Glioma	+	−	[56]
			Lung cancer	+	−	[197]
TAE226	NVP-226	FAK, IGF-IR	Breast cancer	+	+	[198, 199]
			Glioma	+	+	[200, 201]
			Esophageal cancer	+	+	[202, 203]
GSK2256098	–	FAK	Pancreatic cancer	+	−	[204]
			Ovarian cancer	+	−	[205]
PF-431396	–	FAK, Pyk2	Pancreatic cancer	+	+	[196]
			Malignant pleural mesothelioma	+	+	[196]
VS-4718	PND-1186	FAK, Pyk2	Breast cancer/ovarian cancer	+	+	[206]
			Pancreatic cancer	−	+	[123]
Y15	–	FAK	Breast cancer	+	+	[207]
			Lung cancer	+	+	[208]
Y11	–	FAK	Colon cancer/breast cancer	+	+	[176, 204]
C4	–	FAK-VEGFR3 interaction	Breast cancer	+	+	[209]
R2	–	FAK-p53 interaction	CRC	+	+	[210]

**Table 2:** Summary of FAK inhibitors in clinical trials

Name	Alternative names	Target(s)	Cancer types	Status	Phase	NCT number
VS-6063	Defactinib, PF-04554878	FAK, Pyk2	Advanced non-hematologic Malignancies	Completed	1	NCT00787033
			Non-hematologic cancer	Completed	1	NCT01943292
			NSCLC	Completed	2	NCT01951690
			Recurrent skin cancer, squamous cell carcinoma of the skin, stage 0 chronic lymphocytic leukemia, stage I chronic lymphocytic Leukemia	Completed	2	NCT00563290
			Ovarian cancer	Completed	1	NCT01778803
			Solid tumor, pancreatic cancer	Completed	1	NCT02546531
			NSCLC, low grade serous ovarian cancer, endometrioid carcinoma, pancreatic cancer	Recruiting	1	NCT03875820
			Metastatic uveal melanoma	Recruiting	2	NCT04720417
			Pancreas cancer	Recruiting	2	NCT04331041
			Ovarian cancer	Recruiting	1 2	NCT03287271
			PDAC	Recruiting	2	NCT03727880
			Advanced lymphoma, advanced malignant solid neoplasm, hematopoietic and lymphoid cell neoplasm, refractory lymphoma, refractory malignant solid neoplasm, refractory plasma cell myeloma	Active, not recruiting	2	NCT04439331
			Glioma	Not yet recruiting	Early 1	NCT05798507
			Malignant pleural mesothelioma	Terminated	2	NCT02004028
			CRC	Terminated	Early 1	NCT00835679
			Relapsed malignant mesothelioma	Terminated	1	NCT02372227
			NSCLC, mesothelioma, pancreatic neoplasms	Unknown status	1 2	NCT02758587
BI 853520	IN10018, ifebemtinib	FAK	Neoplasms	Completed	1	NCT01335269
			Gastric cancer	Completed	1	NCT05327231
			Metastatic melanoma	Recruiting	1	NCT04109456
			Pancreatic cancer	Recruiting	1 2	NCT05827796
			Platinum-resistant ovarian cancer	Recruiting	1 2	NCT05551507
			Locally advanced or metastatic solid tumor	Active, not recruiting	1 2	NCT05830539
			Solid tumor	Not yet recruiting	1 2	NCT05379946
GSK2256098	–	FAK	Solid tumor	Completed	1	NCT01138033
			Cancer	Completed	1	NCT00996671
			Pancreatic cancer, adenocarcinoma	Completed	2	NCT02428270
			Advanced solid tumor	Completed	1	NCT01938443
			Intracranial meningioma, recurrent meningioma, Nf2 gene mutation	Recruiting	2	NCT02523014
VS-4718	PND-1186	FAK, Pyk2	Pancreatic cancer	Terminated	1	NCT02651727
			Non-hematologic cancer, metastatic cancer	Terminated	1	NCT01849744
			Relapsed or refractory acute myeloid leukemia, relapsed or refractory B-cell acute lymphoblastic leukemia	Withdrawn	1	NCT02215629
CT-707	Conteltinib	FAK, ALK, Pyk2	Advanced pancreatic cancer	Recruiting	1 2	NCT05580445
			NSCLC	Unknown status	1	NCT02695550
VS-6062	PF-00562271	FAK, Pyk2	Head and neck neoplasm, prostatic neoplasm, pancreatic neoplasm	Completed	1	NCT00666926
AMP-945	Narmafotinib	FAK	PDAC	Recruiting	1 2	NCT05355298
APG-2449	–	FAK, ALK, ROS1	Advanced solid cancer, NSCLC, esophageal cancer, ovarian cancer, malignant pleural mesothelioma	Recruiting	1	NCT03917043

**Table 3:** Combination agents of FAK inhibitors

Name	Alternative names	Target(s)	Combination agents	Preclinical trials		Clinical trials	
				Cancer types	References	Cancer types	NCT number
VS-6063	Defactinib, PF-04554878	FAK, Pyk2	Pembrolizumab (a PD1 inhibitor)	–	–	NSCLC, mesothelioma, pancreatic tumor	NCT02758587
				–	–	PDAC	NCT03727880
				–	–	Solid tumor, PDAC	NCT02546531
			VS-6766 (a RAF/MEK inhibitor)	–	–	NSCLC, ovarian cancer, endometrioid carcinoma, pancreatic cancer	NCT03875820
				–	–	Metastatic uveal melanoma	NCT04720417
			Paclitaxel	Ovarian cancer	[189]	Ovarian cancer	NCT01778803
				Pancreatic cancer	[156]	Ovarian cancer	NCT03287271
			Gemcitabine	Lung cancer	[185]	Solid tumor, pancreatic cancer	NCT02546531
			VS-5584 NCT00835679 (a PI3K inhibitor)	–	–	Relapsed malignant mesothelioma	NCT02372227
			Radiation therapy	Pancreatic cancer	[184]	Pancreas cancer	NCT04331041
			Cetuximab	–	–	CRC	NCT00835679
			BRD4 inhibitor	lung cancer	[186]	–	–
			EGFR-TKI	Lung cancer	[187]	–	–
			Docetaxel	Prostate cancer	[188]	–	–
BI 853520	IN10018, ifebemtinib	FAK	Albumin-bound paclitaxel, gemcitabine, KN046	–	–	Pancreatic cancer	NCT05827796
			Cobimetinib, atezolizumab	–	–	Metastatic melanoma	NCT04109456
			PLD	–	–	Platinum-resistant ovarian cancer	NCT05551507
			PLD, toripalimab	–	–	Locally advanced or metastatic solid tumor	NCT05830539
			Docetaxel	–	–	Gastric cancer	NCT05327231
			D-1553	–	–	Solid tumor	NCT05379946
			KRAS G12C inhibitors	CRC, pancreatic cancer, NSCLC	[145]	–	–
			Radiation therapy	Pancreatic cancer	[162]	–	–
			Paclitaxel	Ovarian cancer	[194]	–	–
VS-6062	PF-562,271	FAK, Pyk2	Gemcitabine	Pancreatic cancer	[112]	–	–
			Sunitinib	Hepatocellular cancer	[179]	–	–
			anti-VEGF	Ovarian cancer	[182]	–	–
			AZD-1152	Ewing sarcoma	[183]	–	–
VS-4718	PND-1186	FAK, Pyk2	Nab-paclitaxel, gemcitabine	–	–	Pancreatic cancer	NCT02651727
			Gemcitabine, adoptive cell transfer (ACT), Anti-PD1/anti-CTLA4	Pancreatic cancer	[123]	–	–
GSK2256098	–	FAK	Trametinib (a MEK inhibitor)	–	–	Advanced solid tumor	NCT01938443
			Pazopanib	Ovarian cancer	[205]	–	–
CT-707	Conteltinib	FAK, ALK, Pyk2	Toripalimab, gemcitabine	–	–	Advanced pancreatic cancer	NCT05580445
TAE226	NVP-226	FAK, IGF-IR	Radiation therapy	Glioma	[201]	–	–

### Combination with immunotherapies

Antibodies to immune check point inhibitor that enhance the host immunologic activity against tumors have become standard of care in the treatment of many malignancies [132]. However, only a small percentage of patients have meaningful responses to these treatments. Searching for new pathways and molecules to improve responses and application of immune checkpoint inhibition therapy attracts great attention [133–136]. In TNBC, PD-L1 expression is elevated and significantly correlated with FAK mRNA expression, highlighting the functional relationship between immune checkpoints and FAK [137, 138]. Anti-PD-L1 antibody atezolizumab augments the suppressive impact of FAK inhibitors on cell invasion and migration through the restraint of FAK phosphorylation [138]. Cytokine-induced killer (CIK) cells are used as a treatment approach in adoptive cellular immunotherapy and are highly regarded as a promising candidate for cancer immunotherapy [139]. FAK knockdown/inhibition increased the sensitivity of TNBC cells to CIK cells in coculture system by enhancing CIK-mediated cell death. FAK knockdown also decreased PD-L1 mRNA and protein expression in TNBC cells [140].

Combining the FAK inhibitor VS4718 with anti-PD1 therapy in hepatocellular carcinoma resulted in decreased macrophage numbers and increased CD8+ T-cell numbers [141]. Additionally, when FAK inhibitors were combined with agents that induce T-cell costimulatory pathways in skin squamous cell carcinoma, the tumors became more sensitive to FAK inhibitors, and this effect was mediated by CD80. This suppressed tumor formation and even drove complete regression [118].

Lu et al. constituted a nanodrug PLGA-FAKi by encapsulated FAK inhibitor using poly(lactic-co-glycolic) acid (PLGA). PLGA-FAKi treatment increased ovalbumin-specific CTLs (OVA-CTLs) infiltration into B16-OVA tumors, leading to reduced immunosuppression and increased tumor microvessel permeability, and further inhibited tumor growth when combined with OVA-CTLs [70]. In mice with HGSOC, the combination of FAK inhibitor and anti-TIGIT therapy was able to prolong survival rates, increase the level of CXCL13, which is associated with tumor infiltrating lymphocytes (TLS) formation, and promote B and T-cell enrichment [142]. Mechanical stretching has also been shown to have a positive effect on melanoma cells: it enhances M1 polarization and antitumor effects. This effect is associated with the FAK/NF-kB signaling pathway [143]. It was found that ABCB1, CXCR4, and FAK were overexpressed in non-small cell lung cancer (NSCLC) patients and cell lines [144]. Therefore, targeting CXCR4 and FAK could be a way to overcome DOX resistance and enhance the anti-invasive effects of CXCR4 and FAK inhibitors in NSCLC cells.

### Combination with targeted therapies

Research suggests that co-treatment using KRAS G12C inhibitors and IN10018 is likely to benefit cancer patients with mutated KRAS G12C and may also prevent resistance to KRAS G12C inhibition by targeting dysregulated FAK-YAP signaling and fibrogenesis [145]. Uveal melanoma (UM) patients with unresponsive liver metastases have a druggable downstream signaling hub from GNAQ mutations that activates YAP1 via FAK [146]. Co-targeting FAK and MEK using this approach could lead to novel precision therapy and inhibit tumor growth in UM cells and UM xenograft models. It is important to note that FAK is overexpressed in tumors and activated in iCCA lesions, which in turn contribute to cancer initiation and progression through the YAP proto-oncogene [52]. iCCA growth was dramatically inhibited by combination of FAK and CDK4/6 inhibitor. Overall, the study proved the role of FAK-YAP signaling and suggests its potential as a target for precision therapy in inhibiting cancer growth using various approaches.

Co-targeting this pathway using the FAK inhibitor PF562271 and the BRAF inhibitor vemurafenib could represent a promising therapeutic approach for BRAF-mutant colorectal cancer (CRC) patients, as they exhibit synergistic antitumor effects in vitro and in vivo [147]. Notably, the combination of small-molecule inhibitors of β-catenin or FAK along with vemurafenib not only inhibits the proliferation of BRAF V600E colon cancer cells in vitro but also prevents tumor formation in xenograft mice [148]. Overall, these findings emphasize the potential of combination therapy using several inhibitors in treating cancers with mutations in BRAF and highlights the potential of FAK inhibitors in several therapeutic approaches.

One study found that using TAE226 and sorafenib together effectively reduces hepatocellular carcinoma growth by changing gene expression and epigenetics through FAK nuclear interactome dysregulation [149]. Another study showed that combining VS-6063 and JQ1 to target integrins inhibits FAK signaling and PI3K/AKT, reducing survival in primary HGSOC tumors with co-amplification of FAK and c-Myc [150]. In squamous cell carcinoma cells with mutated FAK, HDAC and FAK inhibitors work synergistically to arrest cellular proliferation and tumor growth, emphasizing the importance of collaborations of multiple targets [151]. In addition, combining SFK/FAK inhibitors with osimertinib shows promise as a therapeutic approach to inhibit growth and resistance in EGFR-mutant lung cancer treatment [152]. Simultaneous targeting of the FAK and Janus kinase/STAT3 pathways produces a synergistic effect. This suggests that repressing STAT3 signals may overcome FAK inhibitor resistance in PDAC, as demonstrated in another study [153]. The studies described above provide evidence that FAK inhibitors, when combined with targeted therapy, present a new and promising avenue of tumor treatment.

### Combination with chemotherapies

Chemotherapy is often ineffective against ovarian cancer; however, the hyaluronic acid-labeled two-in-one drug delivery system HA-PLGA-NPs, containing paclitaxel and FAK siRNA, has high binding efficiency to CD44-positive tumor cells, resulting in increased cytotoxicity and apoptosis in drug-resistant tumors, as demonstrated in experimental studies [154]. FAK inhibition has been identified to enhance chemotherapy sensitivity and promote anticancer effects, primarily through the activation of p53 transcriptional activity, making it a potential focal point for therapeutic strategies in gastric cancer management and a valuable prognostic indicator in clinical settings [155]. Inhibition of FAK, as observed in the study of phosphorylated kinases in PDAC, shows synergistic effects with nab-paclitaxel to reduce tumor growth and appears to be a promising potential treatment option [156]. Furthermore, endothelial cell focal adhesion kinase (EC-FAK) plays a significant role in the regulation of the chemotherapy response and the levels of endocrine factors, and the combination of FAK inhibitors with gemcitabine has the potential to serve as a promising strategy to control PDAC metastasis, as supported by studies that revealed reduced metastasis load and improved survival rates in gemcitabine-treated mice and patients with low levels of EC-FAK [157]. FAK regulates CSC activity in breast cancer, and inhibition of FAK suppresses self-renewal, leading to a reduced tumor size, thereby providing a promising strategy to improve survival by suppressing CSC activity; the approach is especially effective when combined with paclitaxel treatment [98]. In addition, inhibiting endothelial FAK enhances the response of B16 and CMT19T mouse tumors to adriamycin or radiotherapy by suppressing NF-κB activation and cytokine production, thereby improving the effectiveness of DNA damage therapy [158]. Furthermore, elevated EC-PY397-FAK expression levels are strongly correlated with advanced clinical parameters of breast cancer and poor treatment response and independently predict unfavorable five-year recurrence-free survival, which highlights the need to assess the role of FAK inhibitors in optimizing treatments and improve the response to various strategies [159].

### Combination with radiotherapies

Combining FAK inhibitors with low-dose radiation in pancreatic cancer can regulate the TME through several mechanisms, including reducing hypoxia, boosting immune cell infiltration, and enhancing radiosensitivity [160]. FAK overexpression is known to be associated with treatment resistance and metastasis in pancreatic cancer. A database study identified VS-4718 as a potential inhibitor of FAK that can enhance radiosensitivity and inhibit ECM synthesis [161]. Combining FAK inhibition with radiotherapy may prove to be effective against this disease. Tests in a PDAC mouse model have shown that inhibiting FAK with IN10018 can enhance the anticancer effect of radiotherapy by decreasing suppressor granulocyte infiltration and increasing CD8+ T cell and macrophage [162]. This indicates that FAK inhibitors have the potential to enhance the radiosensitivity and immunomodulation of PDAC.

The presence of CSCs in high-grade DCIS is associated with disease recurrence and resistance to radiotherapy via the FAK/Wnt pathway, and the use of FAK inhibitors can reduce cellular self-renewal while enhancing the effects of radiation in breast cancer [163]. Moreover, inhibiting FAK decreased tumor cell adhesion in a glioblastoma/breast cancer cell and endothelial cell coculture model after radiotherapy [164]. The evidence suggests that FAK inhibitors hold promise for enhancing radiosensitivity.

Similarly, a study demonstrated that the combination of FAK inhibition and carbon ion irradiation was effective in inhibiting metastasis in tongue squamous cell carcinoma. The treatment decreased colony formation, increased apoptosis, and reduced migration and invasion in CAL27 cells [165]. Furthermore, in HPV-negative HNSCC cells, FAK inhibition led to enhanced radiosensitivity by inducing G2/M arrest and DNA damage. The study also revealed that lower protein tyrosine kinase 2 (PTK2)/FAK mRNA expression was linked to better disease-free survival. Therefore, PTK2/FAK could be a potential biomarker for HNSCC patients who are susceptible to relapse after radiotherapy [5]. According to the available data, FAK inhibitors have shown promising results in sensitizing cancer cells to the effects of ionizing radiation, which helps reduce tumor burden and recurrence rates.

## Conclusion

Cancer is a complex illness that arises due to diverse genetic and epigenetic alterations that cause abnormalities in multiple biological pathways. Although the development of molecularly targeted therapies has aided in their treatment, their frequent ineffectiveness and drug resistance pose significant challenges owing to recurrence and metastasis s; therefore, new therapeutic targets are urgently needed. Crucial cellular processes such as cell adhesion, migration, proliferation, and survival are regulated by FAK. Furthermore, FAK promotes cancer progression, including features such as tumor angiogenesis, EMT, cancer stemness, and immunomodulatory capacity [166, 167]. FAK is widely activated in multiple cancer types, such as colorectal, lung, ovarian, neck, bladder, breast, and esophageal cancers, and predicts a poor prognosis [98, 168, 169].

The FAK pathway has also been linked to the generation of CSCs [101, 103, 105, 114], which are responsible for tumor propagation, metastasis, and therapy resistance. FAK inhibition has been shown to decrease the number of CSCs, suggesting that FAK may represent a viable target for eliminating CSCs, thereby improving cancer therapy outcomes. In addition, the FAK signaling pathway plays a crucial role in regulating the complex TME [119, 125, 129, 170], which comprises cellular and noncellular components that promote tumor growth and metastasis. Gaining a comprehensive understanding of the involvement of FAK in tumor microenvironment (TME) remodeling is imperative in advancing cancer treatment outcomes to a higher level.

FAK has emerged as a promising target for cancer therapy owing to its key role in tumor cells and the TME. Various FAK inhibitors have demonstrated significant antitumor efficacy in diverse preclinical models and are currently being evaluated in clinical trials. Combining FAK inhibitors with standard cancer treatments has been shown to significantly enhance treatment efficacy and decrease chemotherapy resistance [12, 170], as FAK inhibition sensitizes cancer cells to chemotherapy, leading to better therapeutic outcomes. It is interesting that D-pinitol, a 3-methoxy analogue of d-chiro-inositol in soy foods and legumes, can reduce c-Src kinase activity and NF-kB activation through inhibiting FAK phosphorylation, resulting in decrease of prostate cancer metastasis [171, 172]. Recently, an effective FAK degradation agents have been developed that can selectively degrade FAK and showed outstanding inhibitory effects in triple-negative breast cancer and ovarian cancer cells [173, 174].

However, the clinical translation of FAK inhibitors has been hampered by several challenges. First, a uniform method for measuring the expression of FAK, whether phosphorylated FAK or total FAK, needs to be selected. Second, the selection of an appropriate FAK assay is necessary, and immunohistochemistry, western blotting, and RT‒PCR are the most commonly employed methodologies. Each method has advantages and limitations, and the selection must account for factors such as sensitivity, specificity, and reliability. Finally, there are challenges regarding combination therapies utilizing FAK, including reduced selectivity and specificity, drug resistance, and emerging molecular targets that impact efficacy. As a result, future research should aim to enhance the selectivity and specificity of FAK inhibitors and also develop novel combination therapies to overcome these therapeutic obstacles. By addressing these challenges, the clinical translational impact of FAK-targeted therapies in patients can be optimized, ultimately resulting in more effective and personalized cancer treatments.

## Data Availability

Not applicable.

## Abbreviations

ATE: Atezolizumab
CAFs: Cancer-associated fibroblast
CDK: Cyclin-dependent kinase
CIK: Cytokine-induced killer
CRC: Colorectal cancer
CSCs: Cancer stem cells
ECM: Extracellular matrix
EMT: Epithelial–mesenchymal transformation
EGFR: Epidermal growth factor receptor
FADD: Fas-associated death domain
FAK: Focal adhesion kinase
FAKi: Focal adhesion kinase inhibitor
FAT: Focal adhesion-targeting
FERM: Four-point-one-ezrin-radixin-moesin
HNSCC: Neck squamous cell carcinoma
iCCA: Intrahepatic cholangiocarcinoma
IGF-1: Insulin-like growth factor-1
IGF-1R: Insulin-like growth factor-1 receptor system
JNK: Jun NH2-terminal kinase
LncRNA: Long non‑coding RNA
MaCSC: Mammary cancer stem cells
Mdm2: Murine double minute2
MUCL1: Mucin-like 1
NES: Nuclear export signal
NES2: Nuclear export signal 2
NSCLC: Non-small cell lung cancer
PDAC: Pancreatic ductal adenocarcinoma
PDGF: Platelet-derived growth factor
PDGFR: Platelet-derived growth factor receptor
PTK2: Protein tyrosine kinase 2
PRRs: Proline-rich regions
PyK2: Proline-rich tyrosine kinase 2
RIP: Receptor-interacting protein
RTKs: Receptor tyrosine kinases
Runx1: Runt-related transcription factor 1
SH3: Src homology 3
TLS: Tumor infiltrating lymphocytes
TM4LFM5: Tetraspanin transmembrane 4 L6 family member 5
TME: Tumor microenvironment
TNBC: Triple-negative breast cancer
UM: Uveal melanoma
VEGFR: Vascular endothelial growth factor receptor
VEGFR2: Vascular endothelial growth factor receptor 2
YAP: Yes-associated protein/yes-related protein
